# External Validation of the Modified 4C Deterioration Model and 4C Mortality Score for COVID-19 Patients in a Swiss Tertiary Hospital

**DOI:** 10.3390/diagnostics12051129

**Published:** 2022-05-03

**Authors:** Adriana Wirth, Andrea Goetschi, Ulrike Held, Ataman Sendoel, Melina Stuessi-Helbling, Lars Christian Huber

**Affiliations:** 1 Clinic for Internal Medicine, Department of Internal Medicine, City Hospital Zurich, Triemli, 8063 Zurich, Switzerland; melina.stuessi-helbling@stadtspital.ch (M.S.-H.); lars.huber@stadtspital.ch (L.C.H.); 2 Department of Biostatistics, Epidemiology, Biostatistics and Prevention Institute, University of Zurich, 8001 Zurich, Switzerland; andrea.goetschi@uzh.ch (A.G.); ulrike.held@uzh.ch (U.H.); 3 Institute for Regenerative Medicine, University of Zurich, 8952 Schlieren, Switzerland; ataman.sendoel@uzh.ch

**Keywords:** COVID-19, SARS-CoV-2, hospital, prediction, deterioration, mortality

## Abstract

Prognostic models to predict the deterioration and mortality risk in COVID-19 patients are utterly needed to assist in informed decision making. Most of these models, however, are at high risk of bias, model overfitting, and unclear reporting. Here, we aimed to externally validate the modified (urea was omitted) 4C Deterioration Model and 4C Mortality Score in a cohort of Swiss COVID-19 patients and, second, to evaluate whether the inclusion of the neutrophil-to-lymphocyte ratio (NLR) improves the predictive performance of the models. We conducted a retrospective single-centre study with adult patients hospitalized with COVID-19. Both prediction models were updated by including the NLR. Model performance was assessed via the models’ discriminatory performance (area under the curve, AUC), calibration (intercept and slope), and their performance overall (Brier score). For the validation of the 4C Deterioration Model and Mortality Score, 546 and 527 patients were included, respectively. In total, 133 (24.4%) patients met the definition of in-hospital deterioration. Discrimination of the 4C Deterioration Model was AUC = 0.78 (95% CI 0.73–0.82). A total of 55 (10.44%) patients died in hospital. Discrimination of the 4C Mortality Score was AUC = 0.85 (95% CI 0.79–0.89). There was no evidence for an incremental value of the NLR. Our data confirm the role of the modified 4C Deterioration Model and Mortality Score as reliable prediction tools for the risk of deterioration and mortality. There was no evidence that the inclusion of NLR improved model performance.

## 1. Introduction

Among the patients infected with SARS-CoV-2, the detection of symptoms, clinical signs, and laboratory findings associated with poor outcome is crucial to identify those at high risk of clinical deterioration or death. Although many studies investigating risk factors and prediction models for coronavirus disease 19 (COVID-19) have been published, recent literature indicates that the proposed models are at high risk of bias [1]. Since their performance is probably overestimated, only few of these prediction models are recommended for use in current practice [1,2]. In 2020, two promising risk prediction models for deterioration and mortality in patients hospitalized with COVID-19 were published, based on data from 260 hospitals in England, Scotland, and Wales [3,4]. The authors thoroughly developed and validated these models but suggested their usability for clinical decision making only upon successful validation and potential updating in other settings [5]. The 4C (Coronavirus Clinical Characterisation Consortium) Deterioration Model and Mortality Score have shown to be valid prediction tools for clinical deterioration and in-hospital mortality and outperformed other risk stratification tools [3,4].

The 4C Deterioration Model is a multivariable logistic regression model developed to predict in-hospital clinical deterioration among hospitalized adults with highly suspected or confirmed COVID-19 using 11 variables [3].

The 4C Mortality Score was developed and validated to predict in-hospital mortality in patients with COVID-19 and includes eight parameters routinely available at hospital admission. The score ranges from 0–21 points (Table S1 shows how the score is calculated). Patients with a score of at least 15 had a 62% mortality compared with 1% mortality for those with a score of three or less [4].

Both models showed good discrimination and calibration, but their generalisability remains to be tested. As patients’ characteristics and healthcare systems differ significantly among countries—which might affect the accuracy of prediction—it needs to be determined if the prediction tools also work in populations outside the UK. In this study, we first aimed to externally validate the modified 4C Deterioration Model and 4C Mortality Score in a cohort of Swiss patients. Second, we evaluated whether the inclusion of the neutrophil-to-lymphocyte ratio (NLR) improves the predictive performance of the models, since, as shown by our recent work and others, an elevated NLR identifies COVID-19 patients at risk for clinical deterioration and mortality [6,7]. 

## 2. Methods

### 2.1. Design and Setting

This retrospective single-centre cohort study was performed at the City Hospital Zurich Triemli in Switzerland. For reporting, we adhered to the transparent reporting of a multivariable prediction model for individual prediction or diagnosis (TRIPOD) guidelines [8].

### 2.2. Study Population and Data Collection

Informed consent was obtained from the majority of patients with COVID-19. A surrogate permission (according to Art. 34 HFV) was granted by the cantonal ethics committee Zurich, Switzerland (BASEC-Nr. 2020-01852), for patients from whom no consent could be obtained. The study was conducted in accordance with the declaration of Helsinki.

Adult patients with a positive SARS-CoV-2 swab result using real-time reverse-transcription-polymerase-chain-reaction (RT-PCR) assay (CDC ncov-2019 rT PCR) and have been admitted to our hospital between 27 February and 31 December 2020 were included in our study. Patients transferred from another hospital were excluded for both models if they were treated for more than two days due to COVID-19 prior to transfer. Furthermore, patients were excluded from the 4C Deterioration Model if they met the definition of in-hospital deterioration upon arrival at our hospital. Patients with nosocomial infection were excluded for validation of the 4C Mortality Score [4].

The vital signs were collected separately for the two prediction models: For the Deterioration Model, the first values upon admission were taken. If the SARS-CoV-2 infection was diagnosed later during the hospital stay, we used the values on the day of the positive PCR, as suggested by the authors of the original publication [3]. For the Mortality Score, we used values from hospital admission regardless of whether the patients had been previously hospitalized. For the Deterioration Model, the peripheral oxygen saturation (SpO2) was measured with and without oxygen supplementation. For the Mortality Score, we only used SpO2 breathing room air. If only SpO2 under supplemental oxygen therapy was available, SpO2 was considered below 92%, as suggested by internal guidelines. Results from chest X-ray and CT scans were determined by board-certified radiologists.

### 2.3. Predictors

Predictor definitions were identical to the definitions used for the original model development, except for urea, which was not assessed. For this reason, we refer to both models as ‘modified’. The parameters used for the Deterioration Model are age, sex, nosocomial infection, Glasgow coma scale score (GCS), SpO2 at admission, breathing room air or oxygen therapy (contemporaneous with SpO2 measurement), respiratory rate, C-reactive protein, lymphocyte count, and presence of radiographic chest infiltrates. The parameters used for the Mortality Score are age, sex, respiratory rate, SpO2, GCS, C-reactive protein, and number of comorbidities. Comorbidities (according to the modified Charlson Comorbidity Index [9]) collected were chronic cardiac disease, chronic respiratory disease (excluding asthma), chronic renal disease (estimated glomerular filtration rate ≤30), mild to severe liver disease, dementia, chronic neurological disease, connective tissue disease, diabetes, HIV or AIDS, malignancy, and clinician-defined obesity. If the GCS score on admission was missing, we used descriptions from the medical records to deduce the score of either 15 or below.

In accordance with the methods used in the original publication [3], restricted cubic splines were used to model continuous predictors included in the 4C Deterioration Model. Knot positions were chosen accordingly to Gupta et al.

For the 4C Mortality Score, the same cut-off values as in Knight et al. [4] were used to categorize the continuous variables.

### 2.4. Outcomes

For the 4C Deterioration Model, a composite outcome of in-hospital clinical deterioration was defined, comprising any of the following: the need for ventilator support (high flow therapy, non-invasive ventilation, invasive mechanical ventilation, or extracorporeal membrane oxygenation); admission to a high dependency or intensive care unit; or death [3]. The primary outcome for the 4C Mortality Score was defined as in-hospital death from any cause [4].

### 2.5. Statistical Methods

Descriptive statistics included median and interquartile range for the continuous variables and numbers and percentages of the total for the categorical variables.

### 2.6. Model Validation

For external validation of the modified 4C prediction models, individual risk outcome predictions were made based on the coefficients of the originally published models. We assessed model performance by three different parameters: (i) model discrimination by calculating the area under the receiver operating characteristic curve (AUC) together with a 95% confidence interval (CI) computed as defined by DeLong et al. [10]; (ii) model calibration was inspected visually. Smoothing of the calibration curve was achieved by fitting restricted cubic splines using 4 knots. In addition, the calibration intercept (target value of 0), also termed calibration in-the-large, and slope (target value of 1) together with 95% CIs were estimated; (iii) overall goodness of fit was assessed using the quadratic scoring rule Brier score [11], with lower values indicating better performance. The Brier score depends on the outcome event rate. Therefore, as recommended by Steyerberg et al. [12], the scaled Brier score was additionally calculated, which is scaled by its maximum score under a non-informative model (model that predicts risk to be equal to prevalence for all patients, larger values indicating better performance).

### 2.7. Missing Values

Missing values in one or more of the predictor variables were addressed with multiple imputation based on chained equations [13], under the missingness at random (MAR) assumption. Predictive mean matching using five donors (for each imputation, 1 donor’s observed value is randomly drawn from the 5 candidates to replace the missing value) was used to produce 100 imputed sets (m = 100). The predictors were categorized and transformed after multiple imputation was performed. The distribution of the imputed values for each variable was inspected visually and compared with the distributions of complete cases. For the imputation model, all predictors included in the 4C Deterioration Model and Mortality Score, both outcomes, and additionally dyspnea (y/n), pulmonary disease including asthma (y/n), chronic cardiac disease (y/n), additional infectious disease (y/n), and the disease severity according to the WHO (World Health Organization) classification system [14] were used. The estimates and uncertainty measures from the imputed data sets were combined using Rubin’s rule [15].

### 2.8. Model Recalibration

We compared the outcome incidences in the derivation and our cohorts by simply contrasting the observed prevalence of the outcomes in the Swiss setting to the prevalence of the outcomes in the original setting, in which the models were derived. In order to adjust the two models to the Swiss setting, we updated the baseline risk by re-estimating the models’ intercepts while holding all other coefficients constant. Among others, Morise et al. [16] showed that errors introduced by factors outside the model could potentially be mitigated by this mathematically simple recalibration approach.

### 2.9. Model Updating

The 4C Deterioration Model, as well as the 4C Mortality Score, were both updated by including the additional risk factor NLR. We refrained from categorizing the variable as it was performed for the continuous predictors in the 4C Mortality Score to retain maximal information. Instead, we transformed the variable by taking the natural logarithm to make its distribution less skewed. The other coefficients were held constant, while the coefficient for the variable NLR was estimated.

The three performance measures mentioned above were also calculated for the updated and recalibrated models. After model recalibration, we evaluated whether the predictor NLR should be incorporated in the models by performing likelihood ratio tests as suggested by Vickers et al. [17].

### 2.10. Sensitivity Analysis

As sensitivity analysis, we performed a complete case analysis (all patients for whom the information of one or more predictors was missing were not included). 

## 3. Results

**Figure 1:**
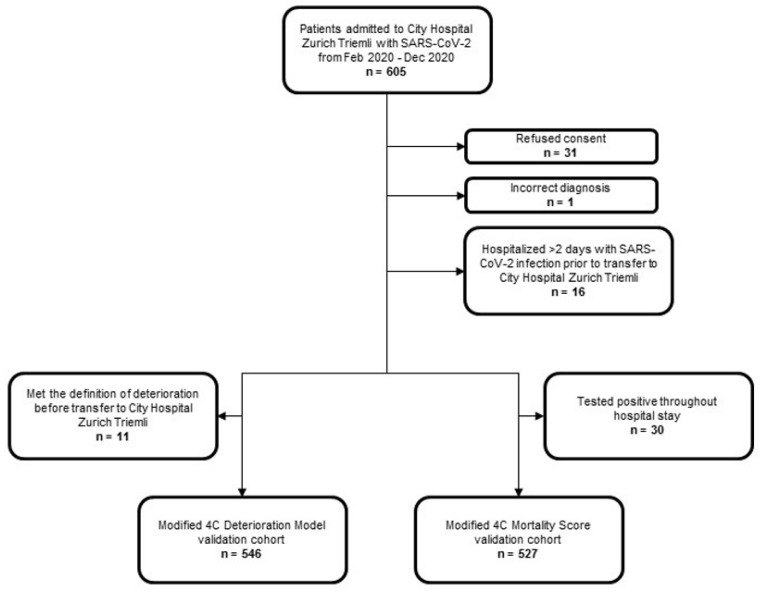
Study population.

Between 27 February and 31 December 2020, 605 patients with positive PCR for SARS-CoV-2 were admitted to City Hospital Zurich Triemli. In total, 31 patients did not consent to the further use of their data. One patient was excluded because the diagnosis was incorrect, and sixteen were hospitalized for more than two days due to COVID-19 in a hospital prior to the transfer to City Hospital Zurich. For the 4C Deterioration Model validation cohort, 11 patients were excluded because they met the definition of deterioration before transfer to our hospital, and for the 4C Mortality Score validation cohort, 30 patients were excluded because they were tested positive throughout the hospital stay. Details are provided in Figure 1.

### 3.1. Modified 4C Deterioration Model

In total, 546 patients (201 females, 345 males) were included in the validation cohort for the modified 4C Deterioration Model. The median age (IQR) was 69 (23) years, compared to 75 (24) years in the original study cohort. Demographic and clinical baseline information on patients included in the main analysis and information assessed throughout the hospital stay are shown in Table 1 stratified by the outcome event (in-hospital deterioration). Baseline characteristics were assessed either at hospital admission or at the time of diagnosis for patients who tested positive for SARS-CoV-2 after the admission date. Predictors were handled identically during modelling as suggested by Gupta et al. (see Table S2).

The outcome was assessed for all patients. In total, 133 (24.4%) patients met the definition of the composite outcome of in-hospital deterioration. The event rate in the original derivation cohort was higher with 43.2%. Moreover, 89 (16.3%) patients were transferred to an ICU, of whom 62 (11.4%) patients needed ventilator support. In total, 59 (10.8%) patients died. Of the 465 patients with complete predictor information, 105 (22.6%) met the definition of in-hospital deterioration.

Discrimination of the 4C Deterioration Model was 0.78 (95% CI from 0.73 to 0.83) (Table 2), which is slightly better than the discriminatory performance of the model when applied to the original derivation cohort (AUC = 0.76 (95% CI from 0.75 to 0.77)). The 4C Deterioration Model overestimated the risk of in-hospital deterioration on average, and the risk predictions varied too little (calibration intercept = −0.39 (−0.60 to −0.18), slope = 1.30 (1.02 to 1.58)) (Figure S1). A slope larger than the reference value 1 suggests that the risk of the outcome event is not extreme enough: patients at high risk of the event tend to receive underestimated risk predictions, whereas patients at low risk of the event tend to receive overestimated risk predictions [18]. As a measure of overall performance, a Brier score of 0.15 was obtained (lower values indicating better performance). The relative performance improvement compared to an uninformative model was 0.19 (larger values indicating better performance). Performance measures did not change considerably after excluding patients with at least one missing predictor information (Table 2).

In order to adjust the model to the Swiss setting, the model was recalibrated by re-estimating the models’ intercept while holding all other coefficients constant. As a result, a pooled model intercept of 3.64 (95% CI from 3.43 to 3.85) was obtained. All performance measurements for the recalibrated model are listed in Table S3.

**Figure 2:**
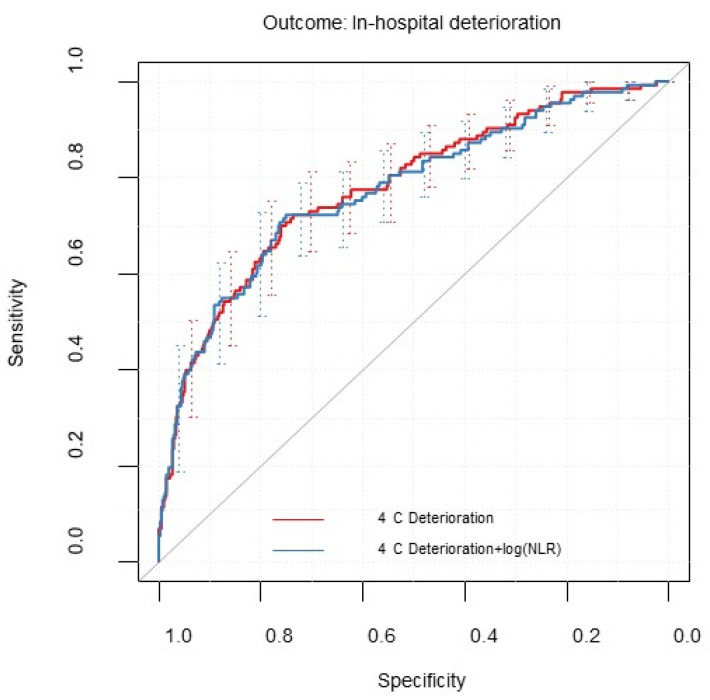
Receiver operating characteristic curve for modified 4C Deterioration Model and updated models including the natural log transformed predictor neutrophil-to-lymphocyte ratio (NLR). Evaluated for the first imputed data set (m = 1) with 95% confidence intervals calculated by bootstrapped resampling (2000 samples).

Updating the model with the additional risk factor NLR did not lead to a considerable change in performance (see Table 2). Figure 2 shows the ROC curves of the 4C Deterioration Model before and after model updating. Calibration was improved after model updating (calibration intercept = −0.12 (−0.33 to 0.10), slope = 1.35 (1.06 to 1.65)). Table 3 shows the pooled log odds ratios of the log-transformed variable NLR and the model intercept estimates.

Comparison between the recalibrated and updated model to the recalibrated model without the additional risk factor NLR did not reveal any evidence that the updated 4C Deterioration model fits the data better than the model without the variable NLR (test statistic: = −1.24, *p*-value = 1).

### 3.2. Modified 4C Mortality Score

A total of 527 patients (196 females, 331 males) were included in the validation cohort for the 4C Mortality Score. The median age (IQR) was 68 (24) years, which is lower than in the original derivation cohort with 76 (25) years. Moreover, 33% of the patients had 2 or more comorbidities compared to 48% in the original derivation cohort. Demographic and clinical baseline information on patients included in the main analysis and information assessed throughout hospital stay are displayed in Table 4 stratified by the outcome event (in-hospital mortality). Predictor cut-offs were chosen according to Knight et al. (see Table S4).

The outcome was assessed for all patients: 55 (10.4%) patients died. The event rate in our cohort was lower than in the original derivation cohort, of which 32.2% of patients died in the hospital.

According to Knight et al., four risk groups with corresponding mortality rates were defined: low risk (0–3 score, mortality rate 1.2%), intermediate risk (4–8 score, 9.9%), high risk (9–14 score, 31.4%), and very high risk (≥15 score, 61.5%). None of the 65 patients belonging to the low-risk group died. We observed an event rate of 2.0% for the 199 patients classified as being at intermediate risk, 17.7% for 254 patients with high risk, and 66.7% for 9 patients in the very high-risk group. Except for the highest category, all mortality rates were lower than the mortality rates used to define the risk groups. 

Discrimination of the 4C Mortality Score was 0.85 (95% CI from 0.79 to 0.89). Surprisingly, the model showed a considerably better discriminatory performance when applied to our cohort than when applied to the original derivation cohort (AUC = 0.79 (95% CI from 0.78 to 0.79)). The obtained estimate for calibration-in-the-large of −1.00 (95% CI from −1.29 to −0.70) implies that, on average, the risk of in-hospital mortality was slightly overestimated. The calibration slope was estimated to be 1.52 (95% CI from 1.10 to 1.94) (Figure S2). As a measure of overall performance, a Brier score of 0.09 was obtained (lower values indicating better performance). The relative performance improvement compared to an uninformative model predicting constant risk was 0.02 (larger values indicating better performance).

When considering only complete cases, the scaled Brier score became negative, indicating a decline in overall performance compared to an uninformative model (see Table 2).

After adjusting the model to the Swiss setting, a pooled model intercept of −5.20 (95% CI from −5.49 to −4.90) was obtained. The overall performance improved marginally after updating the baseline risk (Brier score before and after recalibration: 0.09 vs. 0.08). All performance measurements for the recalibrated model are provided in Table S3.

**Figure 3:**
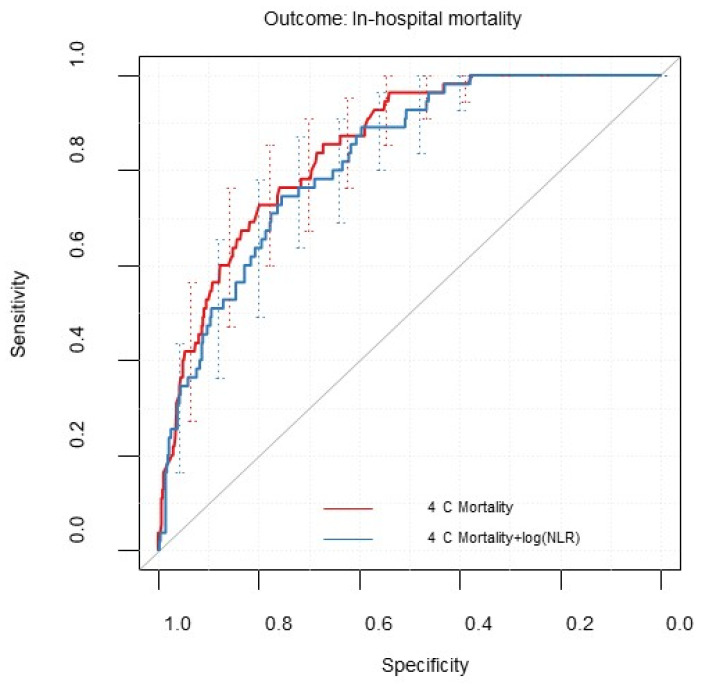
Receiver operating characteristic curve for modified 4C Mortality Score and updated models including the natural log transformed predictor neutrophil-to-lymphocyte ratio (NLR). Evaluated for the first imputed data set (m = 1) with 95% confidence intervals calculated by bootstrapped resampling (2000 samples).

Discrimination declined by updating the model with the additional risk factor NLR (see Table 2). Figure 3 shows the ROC curves of the 4C Mortality Score before and after model updating. After updating the model, the 95% CIs of both calibration measurements included their reference values (calibration intercept = −0.27 (−0.57 to 0.02), slope = 1.35 (0.96 to 1.75)). Table 3 shows the pooled log odds ratios of the log-transformed variable NLR and the model intercept estimates. We compared the recalibrated and updated model to the recalibrated model without the additional risk factor NLR. Similar to the 4C Deterioration Model, there is no evidence for an incremental value of the biomarker NLR (test statistic: = −1.47, *p*-value = 1).

## 4. Discussion

Since the beginning of the pandemic in 2019, more than 107 prognostic models for predicting deterioration and mortality risk for COVID-19 patients have been published [1,19]. However, most of the models are at high risk of bias, model overfitting, and unclear reporting. As such, only a few of them are recommended for use in practice [1].

In this retrospective single-centre analysis, we externally validated the modified 4C Deterioration Model and Mortality Score in patients with laboratory-confirmed SARS-CoV-2 infection. Although the parameter urea was excluded, and despite lower event rates of in-hospital deterioration and mortality in the Swiss setting, both models performed very well and showed good discrimination ability. Before recalibration, the models overestimated the risk of the outcome events on average. To adjust the models to the Swiss setting, we recalibrated both models by updating the baseline risk. We refrained from re-estimating all model coefficients because of the prospect of overfitting. Both models were updated by including the predictor NLR. However, there was no evidence that the inclusion of NLR improved the model fit compared to the original models.

In line with other validation studies [20,21,22,23,24,25], the respective event rate in our cohort was lower than in the original studies. One reason might be that the patients in our cohort were younger than the original cohort. Moreover, the lower median baseline levels of C-reactive protein in our cohorts and the lower percentage of patients with two or more comorbidities indicate that our patient population was less ill. Due to lack of capacity during the first pandemic wave, some patients with severe illness were transferred to other ICUs. In addition, many elderly patients had a healthcare directive documenting that hospitalization or escalation of treatment, including intensive care treatment, should be avoided. The fact that we excluded patients transferred from another hospital and met the criterion of deterioration on hospital admission probably also resulted in a lower event rate in our cohort. Of note, SARS-CoV-2 infection was not the main reason for hospitalization in all our patients. The inclusion of patients, in which SARS-CoV-2 was detected incidentally, might also have affected both outcome scores in our cohort. Finally, whereas for both models’ original derivation, only patients with a minimal follow-up time of 28 days were included, the median (IQR) follow-up time was only 8 (7) days for both cohorts. However, since deterioration after recovery or even following discharge is uncommon in patients with COVID-19, the shorter follow-up period in our patient cohort is unlikely to affect outcome data.

The neutrophil-to-lymphocyte ratio is considered a surrogate marker for outcome and systemic hyperinflammation in patients hospitalized due to COVID-19 [26,27]. As we confirmed recently, baseline NLR not only identifies patients at high risk for deterioration but also accurately differentiates between high and low mortality risk in patients with COVID-19 [6,7]. Surprisingly, we found no evidence for a better model performance when NLR was included as a predictor in both the Deterioration Model and the Mortality Score. These results indicate that the two calculators are excellently powered and that NLR is already represented by other variables included in the models.

Our study has several limitations. The main limitations of this study are the small sample size, the single-centre design and that data collection was performed retrospectively. As such, information on follow-up after hospital discharge and urea levels is lacking. Urea was considered to be of importance in the derivation cohort. Conversely, our data emphasize that the performance of both models is not hampered when urea is excluded from the scoring system. This notion is along the line of other prediction tools that used a similar approach: for predicting the severity of pneumonia, for example, urea measurement was not found to substantially improve the predictive value of the CURB-65 score compared to CRB-65 (without urea) [28,29]. Another limitation is the use of the entire dataset for model recalibration, updating, and subsequent validation. In combination with the limited sample size, such an approach could potentially have led to overfitting.

Our study also has considerable strengths: First, this study represents a comprehensive validation of the 4C Deterioration Model and 4C Mortality Score in a Swiss tertiary hospital setting. Second, we employed state-of-the-art methodology for the validation of prediction models by assessing discrimination (AUC), calibration (intercept, slope), and overall goodness of fit (Brier score). Third, while both scores have already been externally validated in other countries, our data provide the first validation in the Swiss population. Of note, as compared to previous studies, the 4C Mortality Score showed the largest discrimination in the Swiss population (Table 5 and Table 6). In this context, we could show that the two models reliably predict the risk of deterioration and mortality of hospitalized patients with COVID-19 in a representative patient collective of the Swiss population.

## 5. Conclusions

In conclusion, our data highlight that (i) the modified 4C Deterioration Model and Mortality Score performed very well; and (ii) inclusion of urea or NLR appears not to improve the performance of the models. Our findings also indicate that in a setting of limited hospital resources, the modified 4C Deterioration Model and Mortality Score might assist in detecting low-risk patients that can be managed as outpatients and in identifying those at high risk for deterioration. Since these patients should be considered for close monitoring and early transfer to ICU, initial assessment of illness severity is a key part of guiding management and treatment escalation. As we confirm here, both models appear to be useful tools in the process of clinical decision making when treating COVID-19 patients.

## Figures and Tables

**Table 1:** Clinical and demographic characteristics of modified 4C Deterioration Model validation cohort.

Variable	Overall	I.h Deterioration = No	I.h Deterioration = Yes	NA (%)
n	546	413	133	
Male = yes (%)	345 (63.2)	250 (60.5)	95 (71.4)	0.0
Age (years) (median [IQR])	69.00 [56.00, 79.00]	67.00 [53.00, 79.00]	73.00 [62.00, 81.00]	0.0
Respiratory rate (median [IQR])	22.00 [18.00, 27.00]	22.00 [18.00, 26.00]	24.00 [20.00, 30.00]	9.9
SpO2 (%) (median [IQR])	94.00 [91.00, 96.00]	94.00 [92.00, 97.00]	93.00 [89.00, 96.00]	1.1
Oxygen therapy = yes (%)	102 (18.7)	41 (9.9)	61 (45.9)	0.0
Chest infiltrates = yes (%)	416 (76.2)	311 (75.3)	105 (78.9)	0.0
GCS < 15 = yes (%)	38 (7.0)	19 (4.6)	19 (14.3)	0.0
Nosocomial = yes (%)	13 (2.4)	4 (1.0)	9 (6.8)	0.0
CRP (mg/L) (median [IQR])	61.30 [27.40, 118.00]	53.40 [23.00, 102.00]	109.00 [49.70, 154.50]	0.9
Lymphocytes (10^9^/L) (median [IQR])	0.88 [0.60, 1.20]	0.90 [0.65, 1.26]	0.70 [0.50, 0.93]	5.1
NLR (median [IQR])	5.29 [3.29, 8.54]	4.63 [2.85, 7.61]	7.24 [4.77, 13.04]	5.1
LOS (days) (median [IQR])	8.00 [5.00, 12.00]	7.00 [5.00, 11.00]	11.00 [6.00, 22.00]	0.0
Any symptoms = yes (%)	489 (96.4)	370 (96.4)	119 (96.7)	7.1
Symptoms (%)				0.0
Cough	320 (58.6)	248 (60.0)	72 (54.1)	
Fever	280 (51.3)	213 (51.6)	67 (50.4)	
Headache	69 (12.6)	64 (15.5)	5 (3.8)	
Chest pain	73 (13.4)	62 (15.0)	11 (8.3)	
Dyspnoea	233 (42.7)	162 (39.2)	71 (53.4)	
Malaise/Arthralgia/Myalgia	290 (53.1)	233 (56.4)	57 (42.9)	
Nasal congestion	12 (2.2)	11 (2.7)	1 (0.8)	
Gastrointestinal symptoms	114 (20.9)	91 (22.0)	23 (17.3)	
Sore throat	30 (5.5)	24 (5.8)	6 (4.5)	
Anosmia	15 (2.7)	13 (3.1)	2 (1.5)	
Disease severity (%)				0.0
Mild	107 (19.6)	87 (21.1)	20 (15.0)	
Moderate	110 (20.1)	107 (25.9)	3 (2.3)	
Severe	202 (37.0)	195 (47.2)	7 (5.3)	
Critical	127 (23.3)	24 (5.8)	103 (77.4)	
Transfer to ICU (%)	89 (16.3)	0 (0.0)	89 (66.9)	0.0
Interventions on ICU (%)				0.0
Mechanical ventilation	52 (9.5)	0 (0.0)	52 (39.1)	
NIV	10 (1.8)	0 (0.0)	10 (7.5)	
Highflow	1 (0.2)	0 (0.0)	1 (0.8)	
ECMO = no	546 (100.0)	413 (100.0)	133 (100.0)	
Exitus (%)	59 (10.8)	0 (0.0)	59 (44.4)	0.0
Therapy received (%)				0.0
Standard care	249 (45.6)	222 (53.8)	27 (20.3)	
Hydroxychloroquine	50 (9.2)	21 (5.1)	29 (21.8)	
Dexamethasone	35 (6.4)	16 (3.9)	19 (14.3)	
Remdesivir	115 (21.1)	104 (25.2)	11 (8.3)	
Lopinavir/Ritonavir (L/R)	96 (17.6)	50 (12.1)	46 (34.6)	
Dexamethasone & Remdesivir	1 (0.2)	0 (0.0)	1 (0.8)	

Note: I.h = in-hospital; NA = not available; IQR = interquartile range; LOS = length of stay; Fever was defined as temperature *>* 38.5 °C; Disease severity defined according to the WHO classification system [14]; NIV = non-invasive ventilation; ECMO = extracorporeal membrane oxygenation.

**Table 2:** Model performance before and after model updating in SARS-CoV-2 patients admitted to Triemli Hospital.

	AUC	Brier	Brier Scaled	Calibration-in-the-Large	Calibration Slope
	Imputed by MICE				
4C Deterioration	0.781 (0.731 to 0.825)	0.149	0.190	−0.393 (−0.605 to −0.182)	1.298 (1.017 to 1.578)
4C Detrioration + log(NLR)	0.776 (0.724 to 0.821)	0.146	0.207	−0.115 (−0.327 to 0.096)	1.351 (1.057 to 1.646)
4C Mortality	0.846 (0.793 to 0.888)	0.092	0.018	−0.996 (−1.290 to −0.701)	1.521 (1.100 to 1.943)
4C Mortality + log(NLR)	0.823 (0.766 to 0.868)	0.081	0.132	−0.271 (−0.566 to 0.023)	1.353 (0.958 to 1.749)
	Complete case				
4C Deterioration	0.786 (0.736 to 0.837)	0.146	0.164	−0.516 (−0.749 to −0.283)	1.372 (1.049 to 1.694)
4C Detrioration + log(NLR)	0.776 (0.722 to 0.829)	0.141	0.195	−0.145 (−0.376 to 0.086)	1.425 (1.084 to 1.766)
4C Mortality	0.835 (0.779 to 0.890)	0.091	−0.064	−1.124 (−1.454 to −0.795)	1.467 (1.009 to 1.925)
4C Mortality + log(NLR)	0.797 (0.737 to 0.857)	0.079	0.082	−0.335 (−0.663 to −0.007)	1.176 (0.764 to 1.587)

**Table 3:** Pooled neutrophil-to-lymphocyte ratio (NLR) and recalibrated intercept estimates.

	log(Intercept)	log(OR_log(NLR)_)
4C Deterioration_recal_	3.640 (3.428 to 3.851)	-
4C Deterioration + log(NLR)	-	−0.154 (−0.259 to −0.048)
4C Deterioration_recal_ + log(NLR)	3.316 (2.740 to 3.893)	0.175 (−0.112 to 0.461)
4C Mortality_recal_	−5.199 (−5.493 to −4.904)	-
4C Mortality + log(NLR)	-	−0.403 (−0.544 to −0.262)
4C Mortality_recal_ + log(NLR)	−5.681 (−6.461 to −4.900)	0.248 (−0.113 to 0.609)

Note: OR = odds ratio.

**Table 4:** Clinical and demographic characteristics of modified 4C Mortality Score validation cohort.

Variable	Overall	I.h Mortality = No	I.h Mortality = Yes	NA (%)
n	527	472	55	
Male = yes (%)	331 (62.8)	295 (62.5)	36 (65.5)	0.0
Age (years) (median [IQR])	68.00 [55.00, 79.00]	66.00 [53.00, 78.00]	81.00 [73.50, 86.50]	0.0
Number of comorbidities (%)				0.0
0	183 (34.7)	175 (37.1)	8 (14.5)	
1	172 (32.6)	155 (32.8)	17 (30.9)	
≥2	172 (32.6)	142 (30.1)	30 (54.5)	
Respiratory rate (median [IQR])	22.00 [18.00, 27.00]	22.00 [18.00, 26.00]	24.50 [20.00, 30.00]	11.0
SpO2 (%) (median [IQR])	93.00 [89.00, 96.00]	94.00 [90.00, 96.00]	87.50 [81.75, 92.00]	1.9
GCS < 15 = yes (%)	39 (7.4)	26 (5.5)	13 (23.6)	0.0
CRP (mg/L) (median [IQR])	63.80 [27.55, 120.00]	59.70 [26.40, 115.00]	94.60 [49.10, 145.50]	0.8
NLR (median [IQR])	5.25 [3.29, 8.67]	5.00 [3.12, 8.09]	8.17 [5.44, 13.95]	2.7
LOS (days) (median [IQR])	8.00 [5.00, 12.00]	8.00 [5.00, 12.00]	7.00 [3.00, 13.00]	0.0
Any symptoms = yes (%)	477 (97.1)	431 (97.1)	46 (97.9)	6.8
Symptoms (%)				0.0
Cough	321 (60.9)	295 (62.5)	26 (47.3)	
Fever	273 (51.8)	251 (53.2)	22 (40.0)	
Headache	69 (13.1)	68 (14.4)	1 (1.8)	
Chest pain	72 (13.7)	69 (14.6)	3 (5.5)	
Dyspnoea	239 (45.4)	207 (43.9)	32 (58.2)	
Malaise/Arthralgia/Myalgia	285 (54.1)	261 (55.3)	24 (43.6)	
Nasal congestion	13 (2.5)	12 (2.5)	1 (1.8)	
Gastrointestinal symptoms	110 (20.9)	99 (21.0)	11 (20.0)	
Sore throat	30 (5.7)	28 (5.9)	2 (3.6)	
Anosmia	15 (2.8)	13 (2.8)	2 (3.6)	
Disease severity (%)				0.0
Mild	95 (18.0)	84 (17.8)	11 (20.0)	
Moderate	106 (20.1)	106 (22.5)	0 (0.0)	
Severe	194 (36.8)	192 (40.7)	2 (3.6)	
Critical	132 (25.0)	90 (19.1)	42 (76.4)	
Transfer to ICU (%)	90 (17.1)	76 (16.1)	14 (25.5)	0.0
Interventions on ICU (%)				0.0
Mechanical ventilation	58 (11.0)	46 (9.7)	12 (21.8)	
NIV	9 (1.7)	8 (1.7)	1 (1.8)	
Highflow	1 (0.2)	1 (0.2)	0 (0.0)	
ECMO	1 (0.2)	1 (0.2)	0 (0.0)	
Therapy received (%)				0.0
Standard care	235 (44.6)	218 (46.2)	17 (30.9)	
Hydroxychloroquine	48 (9.1)	41 (8.7)	7 (12.7)	
Dexamethasone	40 (7.6)	29 (6.1)	11 (20.0)	
Remdesivir	108 (20.5)	103 (21.8)	5 (9.1)	
Lopinavir/Ritonavir (L/R)	1 (0.2)	1 (0.2)	0 (0.0)	
Dexameth. and Remdesivir	94 (17.8)	79 (16.7)	15 (27.3)	
Hydroxychl. and L/R	1 (0.2)	1 (0.2)	0 (0.0)	

Note: I.h = in-hospital; NA = not available; IQR = interquartile range; LOS = length of stay; Fever was defined as temperature *>* 38.5 °C; Disease severity defined according to the WHO classification system [14]; NIV = non-invasive ventilation; ECMO = extracorporeal membrane oxygenation.

**Table 5:** Deterioration Model validation performance compared to original Model.

Study	Date of Patient Inclusion	AUC (95% CI)	N	Event Rate (%)	Median Age (Years) (IQR)	Male (%)	Country
Gupta et al. [3]	February 2020–August 2020	0.77 (0.76 to 0.78)	8239	45.9	75 (24)	56	UK
Cowan et al. [20]	August 2020–April 2021	0.75 (0.71 to 0.78)	950	29.6	70 (29)	52	UK
City Hospital Zurich	February 2020–December 2020	0.78 (0.73 to 0.82)	546	24.4	69 (23)	63	Switzerland

Note: IQR = interquartile range.

**Table 6:** Mortality Score validation performance compared to original Score.

Study	Date of Patient Inclusion	AUC (95% CI)	N	Event Rate (%)	Median Age (Years) (IQR)	Male (%)	Country	Notes
Knight et al. [4]	Mai 2020–June2020	0.77 (0.76 to 0.77)	22,361	30.1	76 (25)	54	UK	
Van Dam et al. [21]	March 2020–May 2020	0.84 (0.79 to 0.88)	403	23.6	71 (18)	66	Netherlands	
Adderley et al. [22]	January 2020–August 2020	0.75 (0.72 to 0.79)	1040	28.0	68 (18) *	57	UK	
Lazar Neto et al. [23]	February 2020–June2020	0.78 (0.75 to 0.81)	1363	23.5	61 (16) *	59	Spain, Brazil	
Kuroda et al. [24]	January 2020–May 2020	0.84 (0.80 to 0.88)	693	15.6	68 (15) *	65	Japan	only patients with cardiovascular disease
Jones et al. [25]	March 2020–June2020	0.77 (0.79 to 0.87)	959	23.4	72 (24)	55	Canada	
City Hospital Zurich	February 2020–December 2020	0.85 (0.79 to 0.89)	527	10.4	68 (24)	63	Switzerland	

Note: * mean age (SD), SD = standard deviation.

## Data Availability

The data presented in this study are available on request from the corresponding author. The data are not publicly available due to ethical restrictions.

## Supplementary Materials

The following supporting information can be downloaded at: https://www.mdpi.com/article/10.3390/diagnostics12051129/s1. Table S1: 4C Mortality Score for in-hospital mortality in patients with COVID-19 by Knight et al. Table S2: Model parameters for 4C Deterioration Model adopted from Gupta et al. Table S3: Model performance before and after model updating in SARS-CoV-2 patients admitted to Triemli Hospital after model recalibration. Table S4: Model parameters for 4C Mortality Score adopted from Knight et al. Figure S1: Predicted versus observed probability of in-hospital deterioration for the original 4C Deterioration Model. Figure S2: Predicted versus observed probability of in-hospital mortality for the original 4C Mortality Score.
