# Supplementary material for: External Validation of the Modified 4C Deterioration Model and 4C Mortality Score for COVID-19 Patients in a Swiss Tertiary Hospital
Source: Diagnostics (Basel). 2022 May 3;12(5):1129. doi: 10.3390/diagnostics12051129 (PMC9139628; doi:10.3390/diagnostics12051129)

External validation of the modified 4C Deterioration Model and 4C Mortality Score for COVID-19 patients in a Swiss tertiary hospital

Adriana Wirth[1,*], Andrea Goetschi[2], Ulrike Held[2], Ataman Sendoel[3], Melina Stuessi-Helbling[1,¶] and Lars C. Huber[1,¶]

Affiliations

[1] Clinic for Internal Medicine, Department of Internal Medicine, City Hospital Zurich, Triemli, 8063 Zurich, Switzerland

[2] Department of Biostatistics, Epidemiology, Biostatistics and Prevention Institute, University of Zurich, 8001 Zurich, Switzerland

[3] Institute for Regenerative Medicine, University of Zurich, 8952 Schlieren, Switzerland

* Corresponding author, E-mail: adriana.wirth@stadtspital.ch

¶ MSH and LH share Senior Authorship

Supplementary Materials

Table S1. 4C Mortality Score for in-hospital mortality in patients with COVID-19 by Knight et al.

Variable	4C Mortality Score
Age (years)	
<50	-
50-59	+2
60-69	+4
70-79	+6
≥80	+7
Sex at birth	
Female	-
Male	+1
Number of comorbidities	
0	-
1	+1
≥2	+2
Respiratory rate (breaths/min)	
<20	-
20-29	+1
≥30	+2
Peripheral oxygen saturation on room air (%)	
≥92	-
<92	+2
Glasgow coma scale score	
15	-
<15	+2
Urea (mmol/l)	
<7	-
7-14	+1
>14	+3
C reactive protein (mg/l)	
<50	-
50-99	+1
≥100	+2

Table S2. Model parameters for 4C Deterioration Model adopted from Gupta et al.

Characteristic	log(OR)
Intercept	4.033
Age (years)	0.0159
Age (Spline 1)	-0.0129
Age (Spline 2)	0.1265
Sex Male	0.2690
Nosocomial<br>Yes	0.2439
Radiographic infiltrates<br>Yes	0.3252
Respiratory rate (breaths/min)	-0.0145
Respiratory rate (Spline 1)	0.5992
Respiratory rate (Spline 2)	-1.0780
SpO_2 (%)	-0.0707
SpO_2 (Spline 1)	-0.0248
SpO_2 (Spline 2)	1.0240
Room air or oxygen<br>Oxygen therapy	0.7450
Glasgow coma scale score<br><15	0.6028
CRP (mg/L)	0.0097
CRP (Spline 1)	-0.0395
CRP (Spline 2)	0.0588
Lymphocytes (10^9/L)	-0.4564
Lymphocytes (Spline 1)	0.7309
Lymphocytes (Spline 2)	-0.8113

Figure S1. Predicted versus observed probability of in-hospital deterioration for the original 4C Deterioration Model

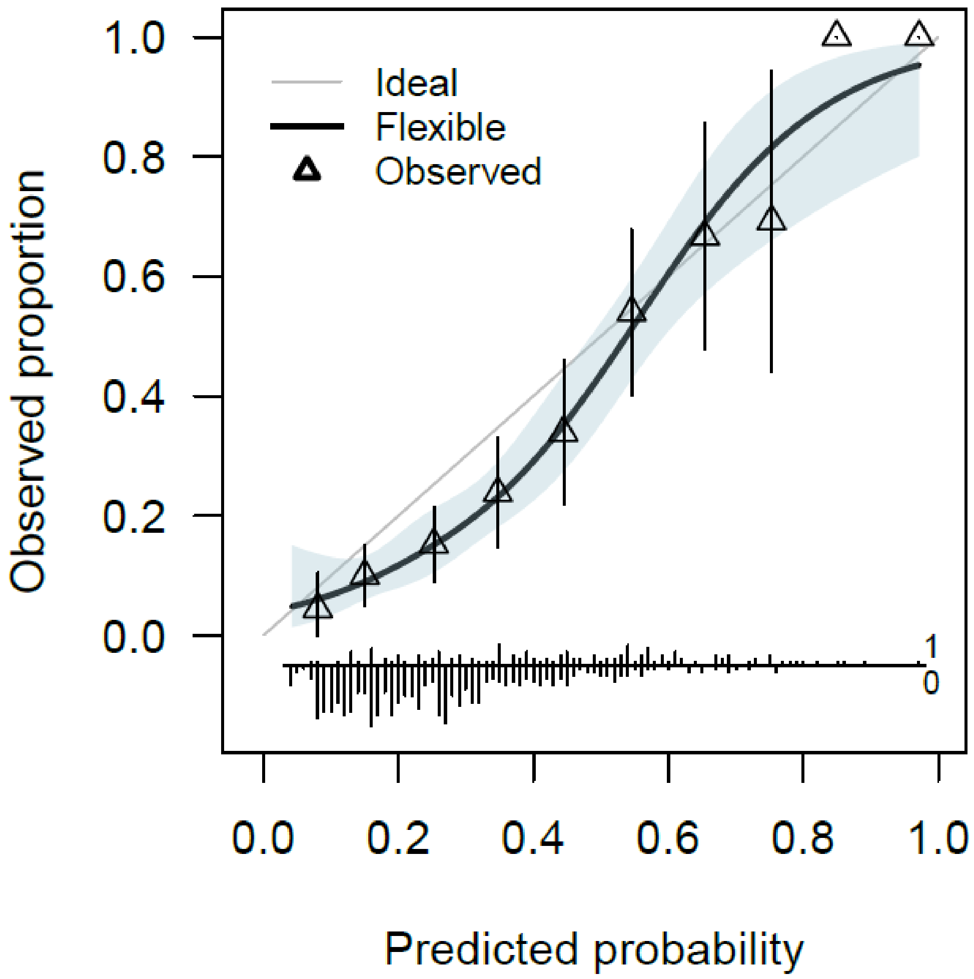

Table S3. Model performance before and after model updating in SARS-CoV-2 patients admitted to Triemli Hospital after model recalibration.

	Imputed by MICE				
	AUC	Brier	Brier scaled	Calibration-in-the-large	Calibration slope
4C Deterioration	0.781 (0.731 to 0.825)	0.145	0.213	0.000 (-0.211 to 0.211)	1.298 (1.017 to 1.578)
4C Detrioration+log(NLR)	0.783 (0.733 to 0.826)	0.145	0.214	0.000 (-0.214 to 0.214)	1.201 (0.939 to 1.464)
4C Mortality	0.846 (0.793 to 0.888)	0.078	0.170	0.000 (-0.294 to 0.294)	1.521 (1.100 to 1.943)
4C Mortality+log(NLR)	0.844 (0.791 to 0.886)	0.077	0.178	0.000 (-0.298 to 0.298)	1.370 (0.990 to 1.750)
	Complete case				
4C Deterioration	0.786 (0.736 to 0.837)	0.139	0.204	0.000 (-0.233 to 0.233)	1.372 (1.049 to 1.694)
4C Detrioration+log(NLR)	0.790 (0.741 to 0.838)	0.139	0.203	0.000 (-0.237 to 0.237)	1.209 (0.923 to 1.495)
4C Mortality	0.835 (0.779 to 0.890)	0.073	0.152	0.000 (-0.329 to 0.329)	1.467 (1.009 to 1.925)
4C Mortality+log(NLR)	0.848 (0.797 to 0.899)	0.071	0.171	0.000 (-0.339 to 0.339)	1.200 (0.846 to 1.555)

Note: MICE = Multiple imputation by chained equations, NLR = neutrophil-to-lymphocyte ratio, Reference values for Brier Scores: Brier max 4C Mortality Score: MICE: 0.093 CC: 0.086 Brier max 4C Deterioration model: MICE: 0.184 CC: 0.175

Table S4. Model parameters for 4C Mortality Score adopted from Knight et al.

Characteristic	log(OR)
Intercept	-4.203
Age (years)	
50-59	0.687
60-69	1.337
70-79	1.842
≥80	2.252
Sex Male	0.172
Number of comorbidities	
1	0.300
≥2	0.532
Respiratory rate (breaths/min)	
20-29	0.232
≥30	0.649
SpO_2 on room air (%)	
<92	0.577
Glasgow coma scale score	
<15	0.558
CRP (mg/L)	
50-99	0.363
≥100	0.740

Figure S2. Predicted versus observed probability of in-hospital mortality for the original 4C Mortality Score

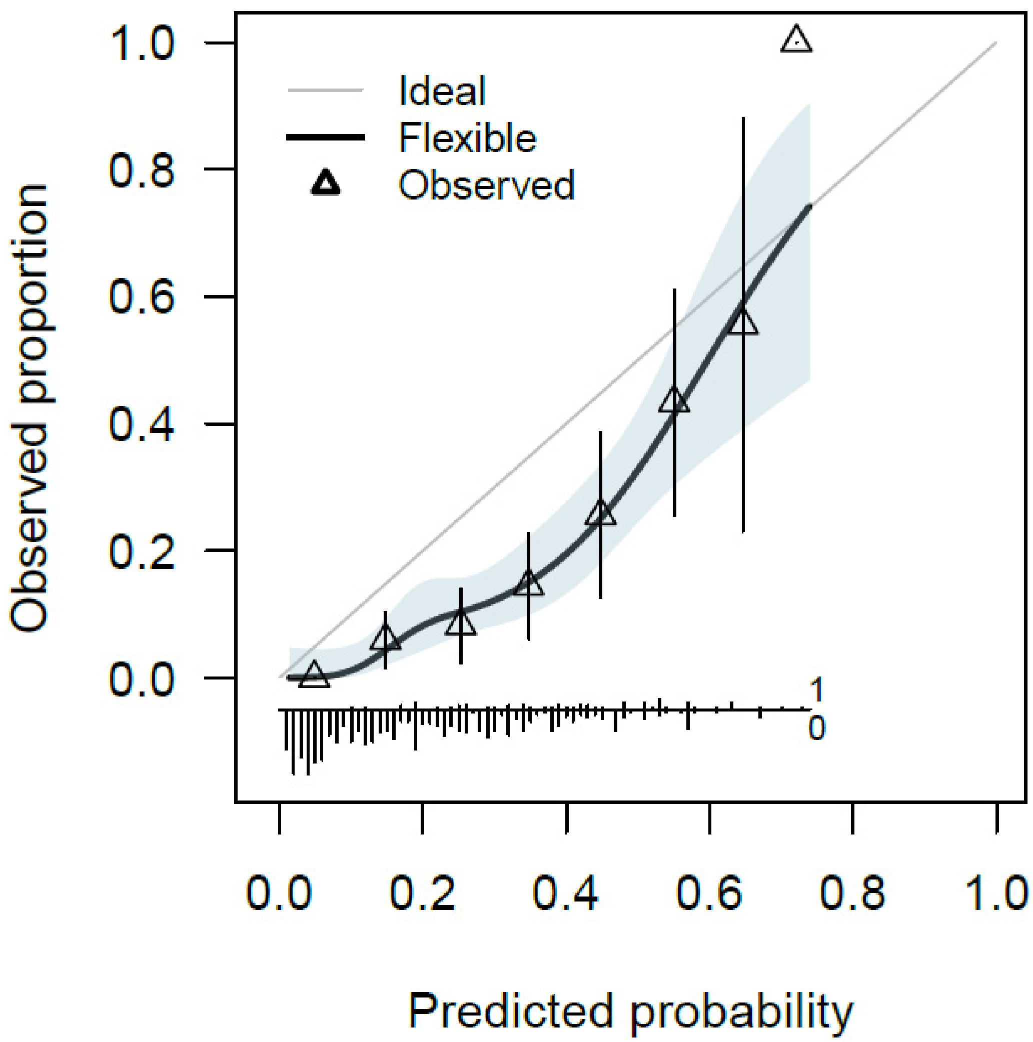

Supplement: Supplementary file 1 [file diagnostics-12-01129-s001.zip › diagnostics-1664572-supplementary.pdf]
